# Editorial: Regulation of metabolic rewiring in T-cell malignancies

**DOI:** 10.3389/fonc.2024.1384469

**Published:** 2024-03-18

**Authors:** Santosh Kumar, Naveen Kumar Vishvakarma, Ajay Kumar

**Affiliations:** ^1^ Department of Life Science, National Institute of Technology (NIT) Rourkela, Sundargarh, Odisha, India; ^2^ Department of Biotechnology, Guru Ghasidas Vishwavidyalaya, Bilaspur, India; ^3^ Tumor Biomarker and Therapeutics Lab, Department of Zoology, Institute of Science, Banaras Hindu University, Varanasi, Uttar Pradesh, India

**Keywords:** T cell malignancies, metabolic rewiring, redox dynamics, miRNAs, cytokines, melatonin

The metabolic aberrations play a pivotal role in the development and progression of malignant T-cells. Understanding the molecular intricacies of these metabolic alterations is essential to better understand the regulatory pathways involved in the disease and to identify novel therapeutic targets for designing promising therapeutic strategies against T-cell malignancies. The Research Topic aims to understand diverse aspects of metabolic alterations in T-cell malignancies mediated by various biomolecules like ROS, bioactive lipids, hormones, amino acids, miRNAs, and cytokines. This Research Topic comprises 7 review articles that discuss the factors involved in changing the metabolic behavior of malignant cells for their uninterrupted growth and progression.

The components of metabolism show direct or indirect association with the growth behavior of neoplastic cells. The benefits of rewired lipid metabolism in tumor growth and adaptation to the tumor microenvironment are beautifully described by Wang et al. They have discussed various aspects of lipid metabolism deregulation, including augmented lipid uptake, fatty acid synthesis and oxidation, and accumulation of cholesterol in unhindered cancer progression. Further, they highlighted the role of altered lipid and cholesterol metabolism in generating signaling molecules, components of biological membrane, and energy sources for rapid growth and aggressive behavior of cancer. This review also discusses the strategy for the therapeutic targeting of altered lipid metabolic pathways of malignant cells to develop promising cancer therapeutic approaches.

T-cell malignancies originate due to aberration in T-cells, one of the key cells of the adaptive immune system. T-cells have their own metabolic arrangements based on their subsets, stages of development, and activation. Malignant cells of T-cell origin also display revamping of metabolic setups to favor their progression and belligerent phenotypes. An elaborate landscape of the role of reprogrammed lipid metabolism in malignant T-cells has been shown in the article by Mehta et al. Rapidly dividing malignant cells require increased *de novo* biosynthesis of fatty acids and other lipid molecules. In their article, they discussed the role of altered lipid metabolism in inducing various malignant properties, including stemness, invasion, metastasis, angiogenesis, chemoresistance, and immunosuppressive environment. The authors have also shed light on the benefits of rewiring lipid metabolism in altered T-cells that include the production of imitable growth-promoting factors such as survival signals, bioenergetic materials, and immunosuppressants, essentially required for their growth and progression. Further, they emphasized that targeting altered lipid metabolic pathways may have several benefits in the clinical management of T-cell lymphoma patients.

Apart from carbohydrate and lipid metabolism, amino acid metabolism also gets altered in malignant cells. The review article by Wang et al. discusses the role of deregulated amino acid metabolism in the metastatic nature of tumor cells. They discussed the metabolic properties and preferences of metastatic cancer cells compared to primary lesions, highlighting the switch to specific metabolic states for colonizing at different distant organs. The review further provides evidence supporting the hyperactivation of amino acid biosynthetic pathways in metastatic cancer cells and explores how this reprogramming orchestrates energy supply and redox homeostasis. Additionally, the article emphasizes the potential of targeting amino acid metabolism for developing novel therapeutic strategies against metastatic cancer.

Metabolic profiles of cancer also affect oxidative balance, their sensing, and regulatory mechanisms. Singh and Maurya in their article, reviewed the role of oxidative stress caused by imbalance in the exogenous and/or endogenous factors in several aspects of cancer development and progression. Oxidative stress alters the intracellular signal transduction pathways caused due to deregulated redox sensors, like nuclear factor-erythroid 2 related factor 2 (Nrf2), phase-II antioxidant enzyme, and NQO1 (NADPH quinone oxidoreductase-1). The role of reactive oxygen species (ROS)-mediated signaling pathways in the growth of T-cell lymphoma has also been discussed in this article.

The metabolism setup is not only determined by the intrinsic necessities of cells but they are also directed by extrinsic factors. Cytokines are one of the major factors affecting the metabolic setup of malignant cells including those of hematological origin. The article by Yadav et al. efficiently discussed the intricate relationship between cytokines, metabolism, and the tumor microenvironment in the context of T-cell lymphoma. It covers the classification and diagnosis of T-cell lymphomas, emphasizing the challenges associated with their identification and treatment. The review also delves into the role of cytokines and hormones as signaling mediators in the interplay between T-cell metabolism and the tumor microenvironment. Furthermore, it highlights the recent advancements in metabolomics and lipidomics, shedding light on the increased understanding of cancer metabolism. The article also explores the molecular mechanisms and consequences of mitochondrial permeability transition, as well as the complexity of p53-mediated metabolic regulation in tumor suppression. Additionally, it discusses the potential therapeutic targets against acute myeloid leukemia and the implications of altered metabolism in drug resistance.

A hefty number of articles also suggest the connection of regulatory elements like miRNAs in cancer. Mondal et al. in their article delineate the critical role of microRNAs (miRNAs) in the diagnosis and prognosis of T-cell lymphoma. Their article emphasizes the importance of accurate and timely diagnosis in improving therapeutic outcomes for aggressive malignancies, particularly T-cell lymphoma through miRNAs. The article discusses the potential of miRNAs as disease-specific markers, highlighting their relevance for liquid biopsy and early disease identification. Furthermore, it delves into the metabolic impact of miRNAs in malignancies of T-cells. The review also underscores the need for further research to identify and validate specific miRNAs for accurate diagnosis and better prognosis.

Targeting the key components of the exclusive metabolic structure of malignant T-cells can be of therapeutic benefit (Mehta et al.; Rai et al.; Mondal et al.). Apart from specific inhibitors of critical enzymes or transporters, biological response modifiers can be suggested to have great therapeutic potential against several cancers, including T-cell lymphoma. The article by Rai et al. has discussed the role of melatonin in metabolic rewiring in T-cell malignancies. This article highlights the pleiotropic effects of melatonin on various cellular pathways, which regulate key metabolic alterations in neoplastic T-cells. The authors recommend exploring the potential synergies between melatonin and current treatment methods such as chemotherapy or immunotherapy, by investigating its impact on T-cell differentiation, activation, and anti-tumor immune responses, and conducting well-controlled clinical trials to evaluate the efficacy and safety of melatonin-based interventions in patients with T-cell malignancies. The article concludes by emphasizing the need to advance our understanding and harness the therapeutic potential of melatonin to improve patient outcomes in the future.

The metabolic rewiring in cancer is quite an interesting and intriguing topic. However, exclusive arrangements in metabolic setup in T-cell malignancies, one of the complex neoplastic diseases with respect to clinical management, are not well explored as other malignancies. This Research Topic presents a collection of articles exploring the rewiring of metabolism, especially in T-cell malignancies. It is expected that readers will find this Research Topic expedient in appraising the significant research area that emerged from metabolic connections with malignancies.

## Author contributions

SK: Writing – review & editing, Writing – original draft. NV: Writing – review & editing, Writing – original draft. AK: Writing – review & editing, Writing – original draft.

